# Deep eutectic solvent-based manganese dioxide nanosheets composites for determination of DNA by a colorimetric method

**DOI:** 10.1186/s13065-023-00922-5

**Published:** 2023-03-12

**Authors:** Jia Xu, Yuan Yang, Juan Du, Hui Lu, Wenqi Gao, Hongjian Gong, HanXiao

**Affiliations:** Institute of Maternal and Child Health, Wuhan Children’s Hospital (Wuhan Maternal and Child Healthcare Hospital), Tongji Medical College, Huazhong University of Science & Technology, 430016 Wuhan, China

**Keywords:** Hexafluoroisopropanol, Deep eutectic solvents, Manganese dioxide nanosheets, Nucleic acid, Colorimetry

## Abstract

**Background:**

Nucleic acid is the carrier of genetic information and the keymolecule in life science. It is important to establish a simple and feasible method for nucleic acid quantification in complex biological samples.

**Methods:**

Four kinds of hydrogen bond acceptors (choline chloride (ChCl), L-carnitine, tetrabutylammonium chloride (TBAC) and cetyltrimethylammonium bromide (CTAB)) were used to synthesize deep eutectic solvents (DESs) with hexafluoroisopropanol (HFIP). DESs based manganese dioxide (MnO2) nanosheets composites was synthesized and characterized. DNA concentration was determined by a UVVis spectrometer. The mechanism of DNA-DES/MnO2 colorimetric system was further discussed.

**Results:**

The composite composed of DES/MnO2 exhibited excellent oxidase-like activity and could oxidize 3,3’,5,5’ -tetramethylbenzidine (TMB) to produce a clear blue change with an absorbance maximum at 652 nm. When DNA is introduced, the DNA can interact with the DES by hydrogen bonding and electrostatic interactions, thereby inhibiting the color reaction of DES/MnO2 with TMB. After condition optimization, ChCl/HFIP DES in 1:3 molar ratio was used for the colorimetric method of DNA determination. The linear range of DNA was 10–130 µg/mL and exhibited good selectivity.

**Conclusion:**

A colorimetric method based on DES/MnO2 was developed to quantify the DNA concentration. The proposed method can be successfully used to quantify DNA in bovine serum samples.

**Supplementary Information:**

The online version contains supplementary material available at 10.1186/s13065-023-00922-5.

## Introduction

Deep eutectic solvents (DESs), an emerging class of environmentally friendly solvents are formed by hydrogen bond acceptors (HBAs) and hydrogen bond donors (HBDs) in an appropriate ratio. The formation of strong hydrogen bonds leads to a melting point lower than that of each individual component [1]. DESs possess numerous excellent properties including low volatility, ease of storage, stable physical and chemical properties, and good biocompatibility [2, 3]. In addition, the physical or chemical properties of DESs can be tuned by selecting HBAs and HBDs species [4, 5]. Owing to their excellent biocompatibility, DESs have been widely applied in the partitioning of biomass, such as proteins and, nucleotides, and for improving the efficiency of enzymatic reactions [6–11].

Nucleic acid, the carrier of genetic information, is a crucial molecule in life sciences. High-purity nucleic acids are the foundation of studies in clinical trials, genomics, food safety and other fields [12, 13]. However, real samples of nucleic acid usually contain impurities such as metal ions and proteins, which interfere with the reliability of the experimental analysis. Consequently, establishing a convenient and simple method for the accurate quantification of nucleic acids in complex biological samples is of great significance. DESs have been used as green substitutes for traditional organic solvents for nucleic acid extraction from aqueous solutions [8, 9, 11, 14]. In addition, Mondal et al. reported the solubility of DNA in DESs and confirmed the chemical and structural stability of DNA after six months of storage in DESs comprising glycerol and ethylene glycol [15]. Sharma et al. reported that hydrogen bonding is the major driving force that promotes the dissolution of DNA in DESs [16]. A recent and promising improvement in DES-based DNA purification approaches is the use of a combination of nanomaterials [2, 17–19].

Manganese dioxide (MnO_2_) is a functional transition metal oxide and its nanosheets have unique properties, such as a high specific surface area and oxidase-mimicking activity [20, 21]. It has been applied in sensing technology [22, 23], cell imaging [24], magnetic resonance imaging [25], and biomedical analysis [26–28]. 3,3’,5,5’-Tetramethylbenzidine (TMB) is a commonly used chromogenic substrate that can change from colorless to blue in the presence of MnO_2_ nanosheets with oxidase-like activity [29]. A colorimetric method based on MnO_2_ nanosheets/TMB has been reported for the detection and quantification of target compounds and biomacromolecules, including glucose, pesticides, metal ions, antibacterial agents, and nucleic acid [14, 20–22, 29, 30].

Hexafluoroisopropanol (HFIP) is a perfluorinated alcohol with a high density and strong hydrophobicity [31]. HFIP has proven to be an excellent HBD for preparation of high-density HFIP-based DESs with various HBAs [4, 32]. At present, HFIP-based DESs have been successfully employed in the purification of pesticides, anthraquinones, and dyes [4, 31, 33], but they are also used as environmental reaction media [34]. In this study, HFIP-based DESs combined MnO_2_ nanosheets were synthesized and employed for the quantification of DNA for the first time. Choline chloride (ChCl) was selected as the HBA to synthesize the DES with HFIP. The DNA quantification procedure was based on the colorimetric reaction between DES/MnO_2_ and TMB. We demonstrated that this method could accurately quantify DNA from bovine serum samples.

## Materials and Methods

### Materials

Cetyltrimethylammonium bromide (CTAB), sodium acetate (NaAc), anhydrous acetic acid, KMnO_4_, NaOH, (NH_4_)_2_SO_4_, K_2_HPO_4_, KH_2_PO_4_, Na_2_CO_3_, Na_2_HPO_4_ and Na_2_SO_4_ were purchased from Sinopharm Chemical Reagent Co., Ltd. (Shanghai, China). Salmon sperm DNA sodium salt, morpholine ethanesulfonic acid (MES), HFIP, ChCl, L-carnitine, tetrabutylammonium chloride (TBAC), and TMB were purchased from Macklin (Shanghai, China). Bovine serum was purchased from Haoyang Biological Manufacture Co., Ltd. (Tianjin, China). All other reagents were of analytical grade and were commercially available. Deionized (DI) water (18.25 MΩ) was used in all the experiments.

### Instrumentation

The surface modification of the obtained DES and DES/MnO_2_ was investigated using a Nicolet 470 fourier transform infraed (FT-IR) spectrometer (Thermo Fisher Scientific, USA) in a KBr pellet at room temperature. Nuclear magnetic resonance (^1^ H NMR) spectra were obtained using an Avance III 400 MHz spectrometer (Bruker, Germany) and the morphology of the MnO_2_ nanosheets was observed using a JEM-2100 transmission electron microscope (TEM) (JEOL, Japan). Thermal gravimetric analysis (TGA) was performed using TG 209F1 (NETZSCH, Germany). A PHI5000 VersaProbe (PHI, Japan) was used for X-ray photoelectron spectroscopy (XPS) analysis. The zeta potential and dynamic light scattering (DLS) were analyzed using a Zeta sizer Nano ZS90 (Malvern, England). Agitation and extraction were performed using an UXI orbital shaker (Huxi, China). The concentration of the DNA solution was determined using a UV-1600PC ultraviolet-visible (UV-Vis) spectrophotometer (XIPU, China). The obtained MnO_2_ nanosheets were dried using an XMTD-8222 vacuum dryer (Jinghong, China). The obtained DES-MnO_2_ was dried in a ZX-LGJ-1 A freeze dryer (Zhixin, China).

### Preparation of DES

Four types of DESs (ChCl/HFIP, L-carnitine/HFIP, TBAC/HFIP, and CTAB/HFIP) were synthesized by stirring a designed amount of HBAs and HFIP in a 150 mL thick-walled pressure-resistant flask at an appropriated temperature until a homogeneous transparent liquid was formed. After optimization, a DES composed of ChCl/HFIP at a 1:3 molar ratio was prepared.

### Preparation of MnO_2_ nanosheets

MnO_2_ nanosheets were synthesized according to a previous reported method [35, 36]. 20 mg KMnO_4_ was accurately weighed and transferred to a 50 mL conical flask, dissolved in 18 mL of DI water, and stirred for 1 h at room temperature. Subsequently, 60 mg of CTAB was added to the flask and stirred continuously until a stable emulsion was formed. Next, 2 mL of 0.1 mol/L MES was poured into the mixture and reacted for 6 h. Finally, the MnO_2_ nanosheets were washed three times with DI water. After centrifuging at 12,000 rpm for 5 min, the MnO_2_ nanosheets were dried at 60 °C under vacuum.

### Preparation of DES/MnO_2_

DES/MnO_2_ was prepared using a previously reported method [14] with some modifications. Briefly, 20 mg of MnO_2_ nanosheets were dispersed in 2 mL of methanol and 0.5 mL of synthesized ChCl/HFIP DES and the mixture was ultrasonicated for 2 h at room temperature. The resulting solution was centrifugated at 5000 rpm for 10 min and washed three times with methanol. Finally, the DES/MnO_2_ solid was collected by vacuum freeze-drying.

### Colorimetric reaction of DES/MnO_2_ and TMB

50 µL of TMB (2 mg/mL) was dissolved in 1800 µL NaAc (pH 4.0). Subsequently, 150 µL DES/MnO_2_ of different concentrations were added to this above mixed solution and shaken on an incubator shaker for 30 min at room temperature. Finally, the resulting solution was measured at 652 nm by UV-Vis spectrometer.

### Colorimetric determination of DNA concentration

Next, 150 µL of DES/MnO_2_ (0.1 mg/mL) was added to 1750 µL NaAc (pH 4.0) aqueous solution. Therefore, 50 µL of DNA solutions with different concentrations was added to the mixed solution. After the addition of 50 µL TMB (2 mg/mL), the mixture was shaken for 30 min at room temperature. Finally, the absorbance of the resulting solution was measured at 652 nm using a UV-Vis spectrometer.

An aqueous solution of 150 µL DES/MnO_2_ (0.1 mg/mL), 50 µL TMB (2 mg/mL), and 1850 µL NaAc (pH 4.0) was prepared to conduct selectivity experiments. Various non-specific proteins, carbohydrates, and salts were selected to replace DNA and were added to the prepared aqueous solution for the DNA selectivity test. The mixture was then shaken for 30 min at room temperature. Finally, the absorbance of the resulting solution was measured at 652 nm using a UV-Vis spectrometer.

## Results and discussion

### Preliminary studies

ChCl, L-carnitine, TBAC, and CTAB were selected as HBA, and HFIP was selected as the HBD. To determine the extraction potential of the proposed DESs for DNA extraction, six inorganic salts ((NH_4_)_2_SO_4_, K_2_HPO_4_, KH_2_PO_4_, Na_2_CO_3_, Na_2_HPO_4_, and Na_2_SO_4_) were used as phase separation inducers. A system of 0.5 mL DES (ChCl/HFIP, L-carnitine/HFIP, TBAC/HFIP, and CTAB/HFIP) and 0.8 g inorganic salts ((NH_4_)_2_SO_4_, K_2_HPO_4_, KH_2_PO_4_, Na_2_CO_3_, Na_2_HPO_4_, and Na_2_SO_4_) were prepared in 5 mL of aqueous solution. The molar ratio of HBAs to the HFIP was 1:2. DNA (10 µg/mL) was added to investigate the extraction performance of the two-phase system. After separating into two phases, the bottom phase was removed and detected at 260 nm using a UV detector. The extraction results are summarized in Table S1. It can be seen that the DES comprising ChCl and HFIP was suitable for DNA extraction.

Figure S1 shows the effect of the ChCl:HFIP molar ratio on DNA extraction. A system involving 0.5 mL DES with different molar ratios (1:1.5, 1:2, 1:3, and 1:4) and 0.8 g Na_2_SO_4_ was prepared in 5 mL of aqueous solution. It was clear that DNA extraction increased with the molar ratio varying from 1:1.5 to 1:3 and thereafter a declined at molar ratio of 1:4. In conclusion, a DES comprising ChCl and HFIP in a 1:3 molar ratio was suitable for DNA extraction.

### Characterization of DES and DES/MnO_2_

FT-IR spectra and ^1^ H NMR were used to characterize the synthesized DESs. As shown in Fig. S2 the stretching vibration peaks of O-H in pure HFIP and ChCl were observed at 3424 cm^− 1^ and 3293 cm^− 1^, respectively, which shifted to a lower wavenumber of 3165 cm^− 1^ in ChCl/HFIP. The shift of the –OH stretching vibration indicated the existence of hydrogen bonding between ChCl and HFIP. In addition, no new peaks were detected, demonstrating that no chemical reaction occurred during DES synthesis. As shown in Fig. S3, the ^1^ H NMR of ChCl/HFIP is as follows: δ 4.60 (s, 1 H), 4.01 (dd, 2 H), 3.52 (m, 2 H), 3.21 (d, 9 H). These results verified that the HFIP/ChCl DES was successfully synthesized.

**Fig. 1:**
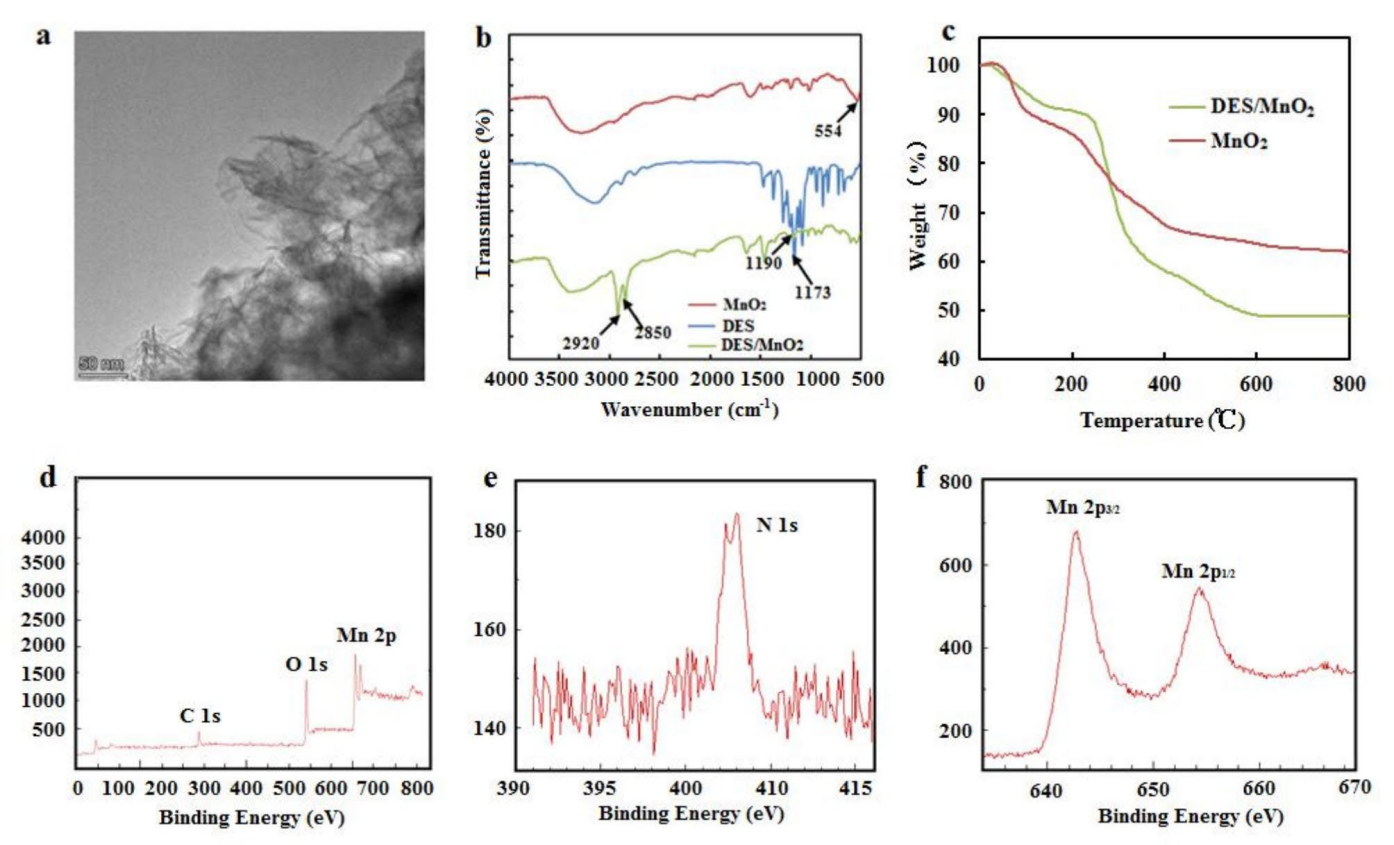
(**a**) The TEM image of MnO_2_ nanosheets; (**b**) FT-IR spectra of MnO_2_ nanosheets, ChCl/HFIP DES and DES/MnO_2_; (**c**) TGA of the MnO_2_ nanosheets and DES/MnO_2_; (**d**) The XPS full scan spectrum of DES/MnO_2_; (**e**) The XPS spectrum of N 1s; (**f**) The XPS spectrum of Mn 2p

The high-resolution TEM image of the prepared MnO_2_ nanosheets (Fig. 1a) revealed the presence of large two-dimensional sheet-like structures, which provided a large surface area for the reaction with TMB, a chromogenic substrate. Figure 1b shows the FT-IR characterization spectrum of the MnO_2_ nanosheets, DES, and DES/MnO_2_, where the MnO_2_ nanosheets exhibited a distinct band at 554 cm^− 1^, which was attributed to Mn-O and Mn-O-Mn. The DES/MnO_2_ spectrum revealed the presence of some characteristic peaks of DES, such as the absorption peaks at 2850 cm^− 1^ and 2920 cm^− 1^, attributed to C-H, and the absorption peaks at 1173 cm^− 1^ and 1190 cm^− 1^ attributed to C-O. These results indicate the successful modification of the MnO_2_ nanosheets by DES. TGA of the MnO_2_ nanosheets and DES/MnO_2_ (Fig. 1c) was performed to determine the mass percentages of the DES in the composites. The decomposition of the DES occurred at 225 °C with a mass loss of approximately 14%, indicating that the DES successfully modified the surface of the nanosheets at a grafting rate of approximately 14%. Figure 1d shows the high-resolution XPS profile of the DES/MnO_2_. The N 1s spectrum (Fig. 1e) confirmed the presence of DES. Moreover, as shown in Fig. 1f, the two characteristic peaks with binding energies of 654.16 eV and 642.68 eV were attributed to the Mn 2p_1/2_ and Mn 2p_3/2_ of MnO_2_, respectively. The XPS spectra also indicated the successful synthesis of DES/MnO_2_.

**Fig. 2:**
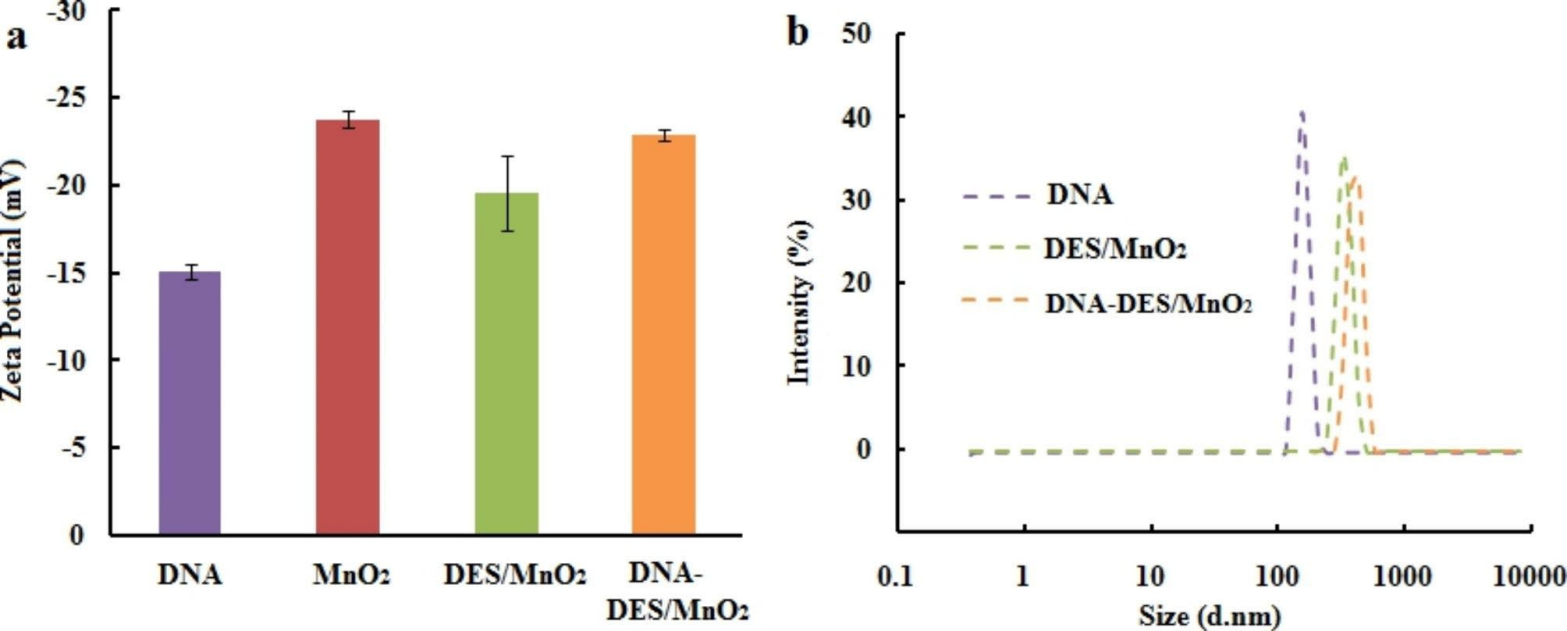
Zeta potentials of DNA, MnO_2_, DES/MnO_2_ and DNA-DES/MnO_2_ (**a**), and sizes distribution of DNA, DES/MnO_2_ and DNA-DES/MnO_2_ (**b**)

DLS and zeta potential measurements were used to investigate the mechanism underlying the detection of DNA by DES/MnO_2_. The zeta potential of the pure DNA, MnO_2_ nanosheets, DES/MnO_2_ and DNA-DES/MnO_2_ were recorded. As shown in Fig. 2a, the zeta potential of the MnO_2_ nanosheet was − 23.77 mV. After combining with DES, the zeta potential of DES/MnO_2_ was approximately − 19.57 mV, which is slightly higher than that of the pure MnO_2_ nanosheets. It was proven that the HFIP/ChCl DES was positively charged. Thus, the negatively charged DNA can bind to the DES through electrostatic interactions and thereafter adsorb onto the surface of DES/MnO_2_. In addition, HFIP contains a large number of hydroxyl groups and is selected as the HBD in the synthesis of DES, which can enhance the hydrogen bond interaction between DES/MnO_2_ and DNA. Therefore, the surface zeta potential of DNA-DES/MnO_2_ was − 22.9 mV, which is slightly lower than that of DES/MnO_2_. Figure 2b shows the DLS results. The particle size of the DES/MnO_2_ was approximately 342 nm. After combining with DNA, the size of the new aggregates was 459 nm, indicating that DNA-DES/MnO_2_ was formed.

### Measurement of the DES/MnO_2_ oxidase activity

**Fig. 3:**
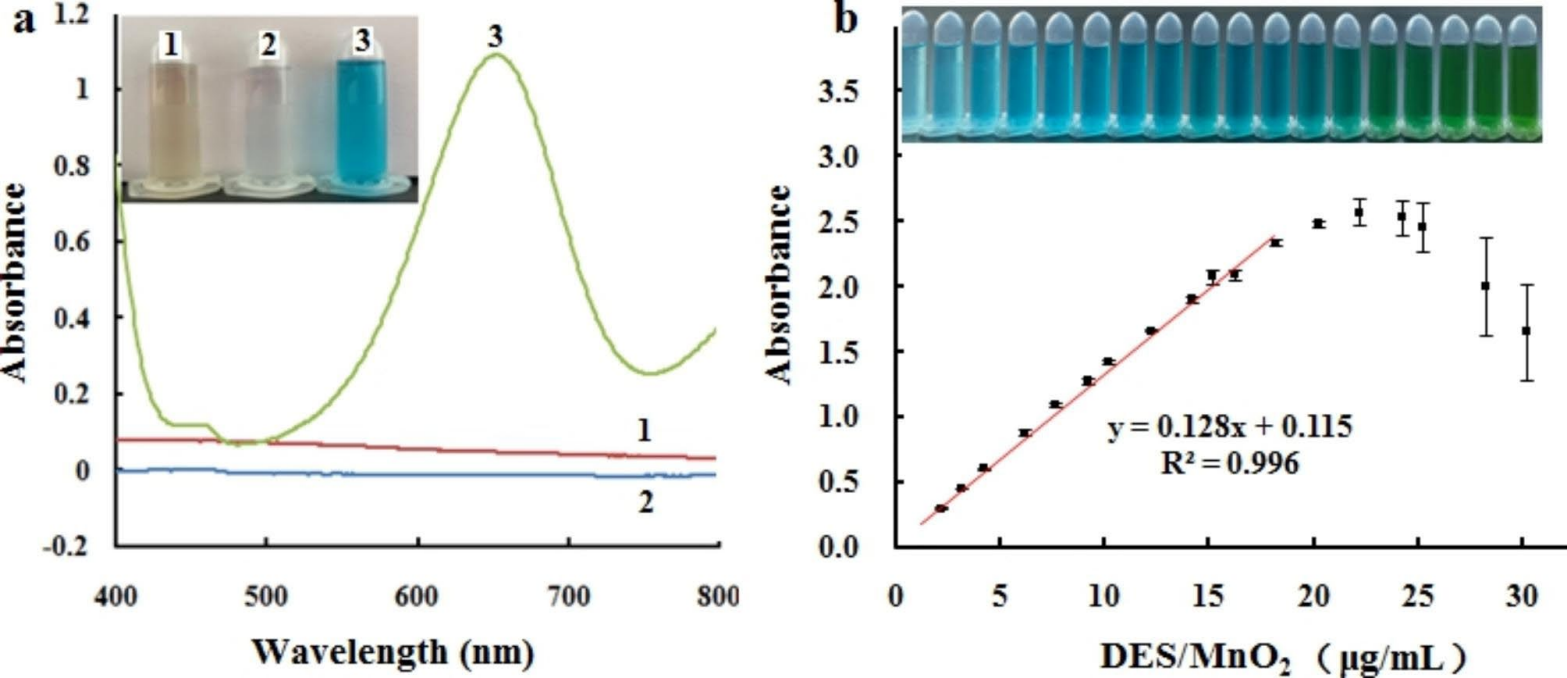
UV absorption spectra of DES/MnO_2_ (line 1), TMB (line 2), DES/MnO_2_ + TMB (line 3) and inset show the corresponding solution color (**a**); the absorbance intensity of DES/MnO_2_-TMB system at different concentrations of DES/MnO_2_ and inset shows the corresponding visual changes in color (**b**). All the error bars were calculated by three independent experiment (n = 3)

TMB was selected as the substrate to investigate the oxidase activity because DES/MnO_2_ possess an oxidase-like activity and can directly oxidize TMB into oxidized TMB (oxTMB). Figure 3a shows neither a significant absorption peak (red) for DES/MnO_2_ nor a significant absorption peak for TMB from 400 to 800 nm (blue). However, owing to the oxidase-like activity of the MnO_2_ nanosheets, a deep blue color (characteristic absorption peak at 652 nm) was observed upon the binding of DES/MnO_2_ with TMB owing to the oxidation of the colorless TMB.

To verify the catalytic activity of DES/MnO_2_ further, different concentrations of DES/MnO_2_ (0–30 µg/mL) were reacted with TMB. The absorbance gradually increased with increasing DES/MnO_2_ concentration (Fig. 3b). However, the absorption intensity decreased when the concentration of DES/MnO_2_ was higher than 22 µg/mL, because TMB or oxTMB may have been denatured. Figure 3b shows a series of color changes. Furthermore, the absorbance signal increased linearly with an increase in the DES/MnO_2_ concentration in the range of 0–18 µg/mL, and the linear regression had an equation of y = 0.128x + 0.115 (R^2^ = 0.996).

**Fig. 4:**
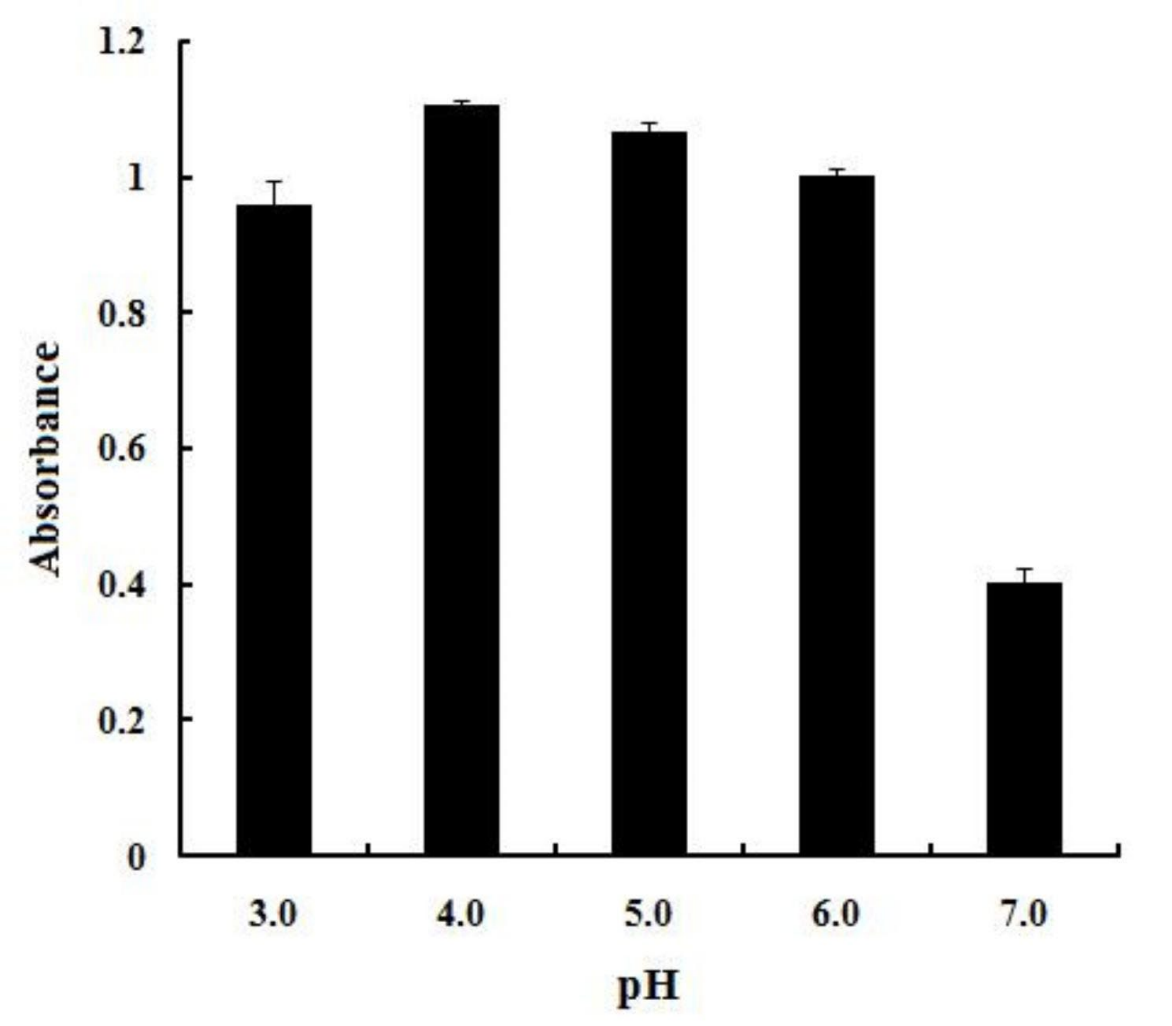
Effect of the pH

The colorimetric reaction of the DES/MnO_2_ composites with TMB under different pH conditions was thereafter evaluated (Fig. 4), and the strongest absorbance response was detected at pH 4.0, which was selected as the optimal pH.

### Colorimetric determination of DNA concentration

**Fig. 5:**
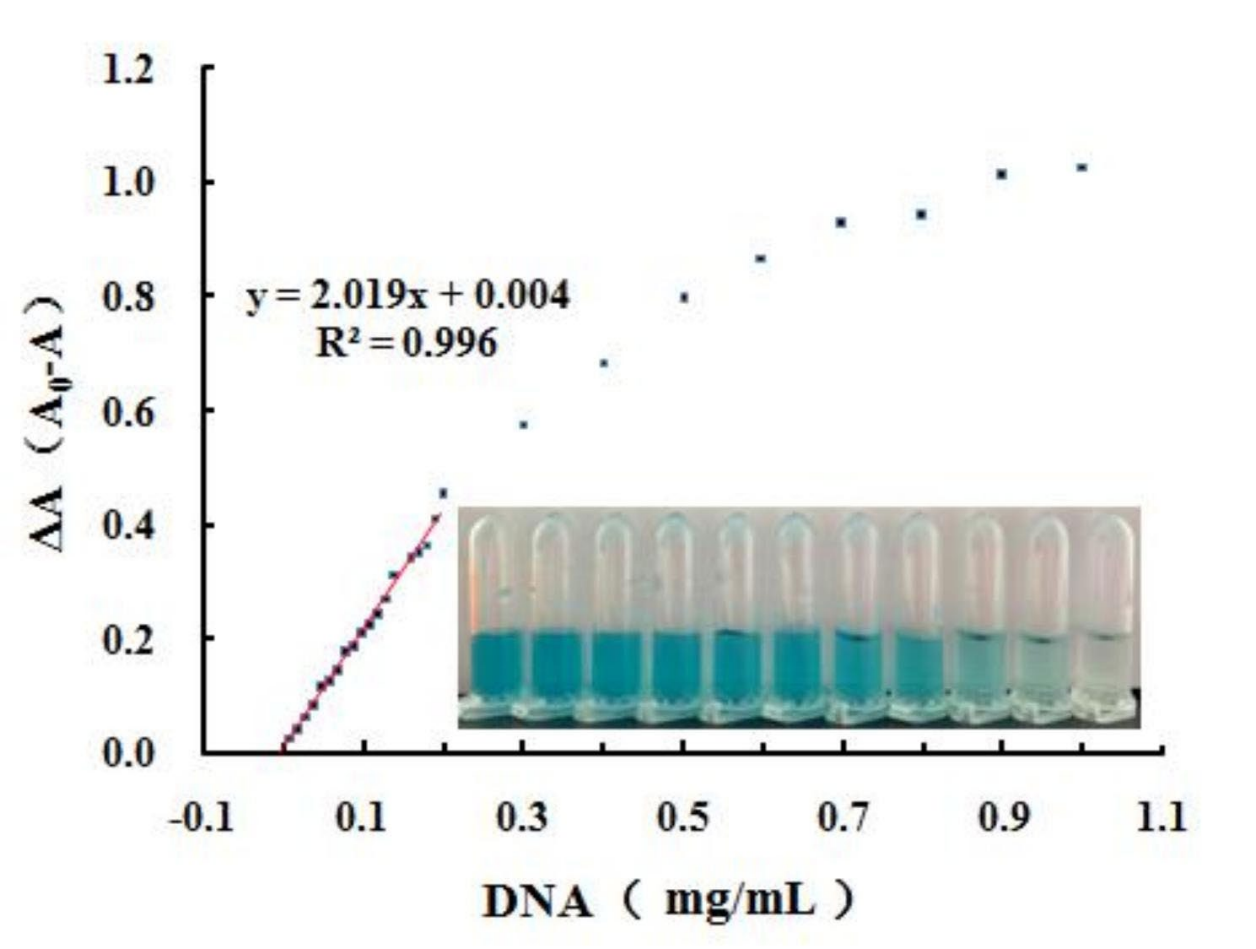
Absorbance intensity of DES/MnO_2_-TMB system at different concentrations of DNA and inset show the color change photographs

To explore the utility of the DES/MnO_2_-TMB system, a colorimetric quantitative analysis of DNA was performed under optimal conditions, and a standard curve was plotted. The difference in absorbance increased with an increase in DNA concentration until it eventually reached a plateau (the image depicts the gradual lightening of the solution color) (Fig. 5). Furthermore, the absorbance difference (ΔA), where ΔA denotes the difference in absorbance of the DES/MnO_2_-TMB system before (A_0_) and after (A) the addition of DNA, exhibited a good linear relationship with DNA concentration in the range of 10–130 µg/mL, and the linear equation was y = 2.019x + 0.004 (R^2^ = 0.996). The adsorption of DNA onto the surface of DES/MnO_2_ was mainly attributed to electrostatic interactions and hydrogen bonding between the phosphate group of DNA and the cationic part of the DES. With the addition of DNA adsorbed on the surface of DES/MnO_2_, the colorimetric reaction of DES/MnO_2_ with TMB was inhibited [21].

### Specificity

**Fig. 6:**
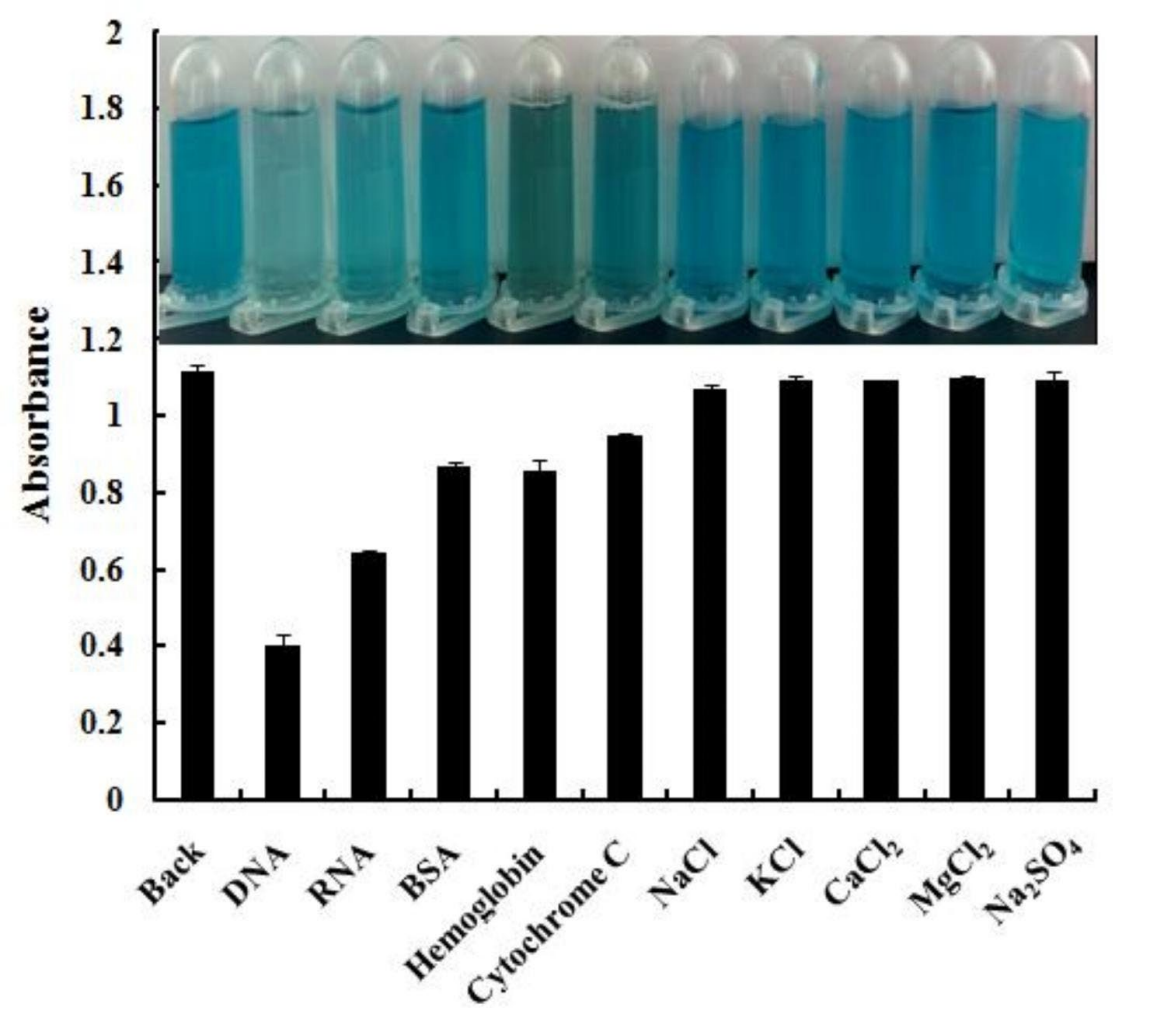
Effect of interfering factors on DES/MnO_2_ + TMB and inset show the color change photographs of DES/MnO_2_-TMB system with different interfering factors

To investigate the specificity of this method for DNA detection, the absorption spectral response of the DES/MnO_2_-TMB system to various interfering substrates (non-specific proteins, carbohydrates, and salts) was studied (Fig. 6). The first column shows the absorption intensities of the DES/MnO_2_-TMB system without the addition of DNA or other interfering substances. RNA had a greater effect on the absorption intensity, whereas proteins such as bovine serum albumin (BSA), hemoglobin, and cytochrome C had a weaker effect. This is primarily because RNA has a structure similar to that of DNA, resulting in a similar inhibitory effect. Consequently, when testing samples containing both DNA and RNA, masking or pre-treatment steps are required.

### Application to real samples

To evaluate the viability of our designed assay for practical applications, DES/MnO_2_ was used to detect DNA in bovine serum. Different concentrations of DNA standard solution were spiked into the bovine serum samples to examine the recovery. The analytical results are summarized in Table 1. The recoveries were within the range of 102.73-107.08% for the three known concentrations of added DNA, and the relative standard deviation (RSD) was less than 3.63%. These results demonstrate the potential application of the proposed colorimetric method for the detection of DNA in real samples.

**Table 1:** Determination of DNA in real sample of bovine serum (n = 3)

Added DNA (µg/mL)	Detected DNA (µg/mL)	Recovery (%)	RSD (%)
20.00	21.42	107.08	3.63
60.00	61.29	102.16	2.40
100.00	102.73	102.73	2.04

## Conclusion

Herein, we report the synthesis of a DES/MnO_2_ composite that efficiently catalyzes TMB. The composition and molar ratio of DESs were evaluated and DES composed of ChCl and HFIP with molar ratio of 1:3 was suitable for DNA extraction. The addition of DNA to the system significantly inhibited the colorimetric reaction and reduced the absorbance of DES/MnO_2_-TMB owing to hydrogen bonding and electrostatic interactions between DNA and the DES. Consequently, a colorimetric method based on DES/MnO_2_ was developed to quantify the DNA concentration. This method exhibited good linearity and specificity and could be used to determine DNA concentration in a simple and rapid manner. Consequently, it exhibits potential for application in DNA detection.

## Electronic supplementary material

Below is the link to the electronic supplementary material.

Table S1 The extraction efficiency of DESs with inorganic salts for the DNA extraction. Fig. S1 The effect of ChCl:HFIP molar ratio on DNA extraction. Fig. S2 FT-IR spectra of ChCl/HFIP DES. Fig. S3 ^1^H NMR spectra of ChCl/HFIP DES. Table S2 The data for Fig. 6.

## Data Availability

The datasets used and/or analyzed during the current study are available from the corresponding author on reasonable request.

## Abbreviations

DESs: Deep eutectic solvents
MnO_2_: Manganese dioxide
TMB: 3,3’,5,5’-tetramethylbenzidine
HBAs: Hydrogen bond acceptors
HBDs: Hydrogen bond donors
DNA: Deoxyribonucleic acid
ChCl: Choline chloride
HFIP: Hexafluoroisopropanol
CTAB: Cetyltrimethylammonium bromide
NaAc: Sodium acetate
MES: Morpholine ethanesulfonic acid
UV-Vis: Ultraviolet-visible
NMR: Nuclear magnetic resonance
TEM: Transmission electron microscope
DLS: Dynamic light scattering
TGA: Thermal gravimetric analysis
XPS: X-ray photoelectron spectroscopy
oxTMB: Oxidized TMB
BSA: Bovine serum albumin.
