# Supplementary material for: Deep eutectic solvent-based manganese dioxide nanosheets composites for determination of DNA by a colorimetric method
Source: BMC Chem. 2023 Mar 12;17(1):15. doi: 10.1186/s13065-023-00922-5 (PMC10010034; doi:10.1186/s13065-023-00922-5)
Supplement: Supplementary file 1 — Table S1 The extraction efficiency of DESs with inorganic salts for the DNA extraction. Fig. S1 The effect of ChCl:HFIP molar ratio on DNA extraction. Fig. S2 FT-IR spectra of ChCl/HFIP DES. Fig. S3 1H NMR spectra of ChCl/HFIP DES. Table S2 The data for Fig. 6. [file 13065_2023_922_MOESM1_ESM.docx]

**Deep eutectic solvent-based manganese dioxide nanosheets composites for determination of DNA by a colorimetric method**

Jia Xu^*^, Yuan Yang, Juan Du, Hui Lu, Wenqi Gao, Hongjian Gong and Han Xiao^*^

（Wuhan Children's Hospital, Tongji Medical College, Huazhong University ofScience & Technology, Wuhan, 430016, China）

* Corresponding author: Jia Xu and Han Xiao

E-mail address: xujia0113@hust.edu.cn (J. Xu), tjxiaohan@hust.edu.cn (H. Xiao)

Table S1 The extraction efficiency of DESs with inorganic salts for the DNA extraction

| HBD | HBA | Inorganic salts | | | | | |
| --- | --- | --- | --- | --- | --- | --- | --- |
|  |  | (NH_4_)_2_SO_4_ | K_2_HPO_4_ | KH_2_PO_4_ | Na_2_CO_3_ | Na_2_HPO_4_ | Na_2_SO_4_ |
| HFIP | ChCl | √ | × | × | × | × | √ |
|  | L-carnitine | × | × | × | - | - | × |
|  | TBAC | × | × | × | × | × | × |
|  | CTAB | × | × | × | - | - | × |

√ The extraction efficiency of DNA was more than 80%.

× The extraction efficiency of DNA was less than 50%.

-The systems cannot be separated into two phases.


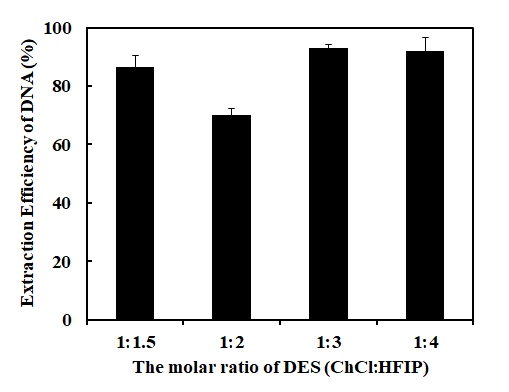


Fig. S1 The effect of ChCl:HFIP molar ratio on DNA extraction.


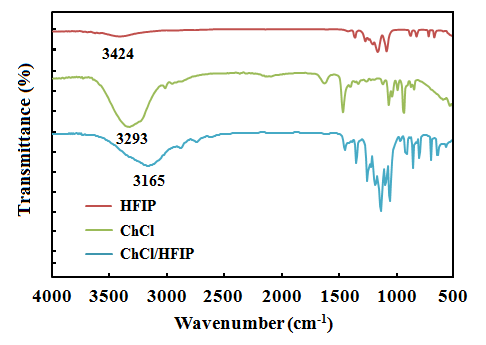


Fig. S2 FT-IR spectra of ChCl/HFIP DES.


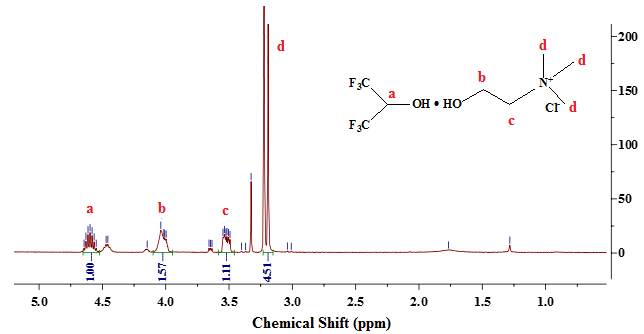


Fig. S3 ^1^H NMR spectra of ChCl/HFIP DES.

Table S2 The data for Fig. 6.

| DNA concentration（mg/mL）  X axis | Absorbance | | | | | |
| --- | --- | --- | --- | --- | --- | --- |
|  | 1 | 2 | 3 | AVERAGE  (A) | SD | △A=A_0_-A  Y axis |
| 0 | 1.0982 | 1.0894 | 1.0835 | 1.0904 (A_0_) | 0.0074 |  |
| 0.01 | 1.0433 | 1.0720 | 1.0749 | 1.0634 | 0.0175 | 0.0270 |
| 0.02 | 1.0512 | 1.0588 | 1.0406 | 1.0502 | 0.0091 | 0.0402 |
| 0.03 | 1.0460 | 1.0174 | 1.0136 | 1.0257 | 0.0177 | 0.0647 |
| 0.04 | 1.0175 | 1.0000 | 1.0062 | 1.0079 | 0.0089 | 0.0825 |
| 0.05 | 0.9730 | 0.9864 | 0.9732 | 0.9775 | 0.0077 | 0.1129 |
| 0.06 | 0.8539 | 1.0274 | 1.0147 | 0.9653 | 0.0967 | 0.1251 |
| 0.07 | 0.9302 | 1.0089 | 0.9073 | 0.9488 | 0.0533 | 0.1416 |
| 0.08 | 0.9606 | 0.8215 | 0.9644 | 0.9155 | 0.0814 | 0.1749 |
| 0.09 | 0.9209 | 0.8869 | 0.9102 | 0.9060 | 0.0174 | 0.1844 |
| 0.1 | 0.9074 | 0.8488 | 0.8914 | 0.8825 | 0.0303 | 0.2079 |
| 0.11 | 0.8495 | 0.8228 | 0.9276 | 0.8666 | 0.0545 | 0.2238 |
| 0.12 | 0.8298 | 0.8225 | 0.8948 | 0.8490 | 0.0398 | 0.2414 |
| 0.13 | 0.7590 | 0.8452 | 0.8542 | 0.8195 | 0.0526 | 0.2709 |
| 0.14 | 0.7544 | 0.7474 | 0.8297 | 0.7772 | 0.0456 | 0.3132 |
| 0.16 | 0.7609 | 0.7765 | 0.7152 | 0.7509 | 0.0319 | 0.3395 |
| 0.17 | 0.7718 | 0.7206 | 0.7287 | 0.7404 | 0.0275 | 0.3500 |
| 0.18 | 0.6642 | 0.7633 | 0.7490 | 0.7255 | 0.0536 | 0.3649 |
| 0.19 | 0.6969 | 0.6809 | 0.6549 | 0.6776 | 0.0212 | 0.4128 |
| 0.2 | 0.6455 | 0.6479 | 0.6254 | 0.6396 | 0.0124 | 0.4508 |
| 0.3 | 0.5026 | 0.5196 | 0.5177 | 0.5133 | 0.0093 | 0.5771 |
| 0.4 | 0.4054 | 0.4062 | 0.4053 | 0.4056 | 0.0005 | 0.6848 |
| 0.5 | 0.2912 | 0.3016 | 0.2934 | 0.2954 | 0.0055 | 0.7950 |
| 0.6 | 0.2381 | 0.2041 | 0.2287 | 0.2236 | 0.0176 | 0.8668 |
| 0.7 | 0.1574 | 0.1468 | 0.1923 | 0.1655 | 0.0238 | 0.9249 |
| 0.8 | 0.1551 | 0.1736 | 0.1224 | 0.1504 | 0.0259 | 0.9400 |
| 0.9 | 0.0889 | 0.0708 | 0.0802 | 0.0800 | 0.0091 | 1.0104 |
| 1 | 0.0620 | 0.0663 | 0.0613 | 0.0632 | 0.0027 | 1.0272 |
